# Growth and survival characteristics of *spa* mice

**DOI:** 10.1002/ame2.12137

**Published:** 2020-10-10

**Authors:** Joline E. Brandenburg, Matthew J. Fogarty, Gary C. Sieck

**Affiliations:** ^1^ Department of Physical Medicine and Rehabilitation Mayo Clinic College of Medicine Rochester MN USA; ^2^ Department of Pediatric and Adolescent Medicine Mayo Clinic College of Medicine Rochester MN USA; ^3^ Department of Physiology and Biomedical Engineering Mayo Clinic College of Medicine Rochester MN USA; ^4^ School of Biomedical Sciences The University of Queensland Brisbane Australia; ^5^ Department of Anesthesiology Mayo Clinic College of Medicine Rochester MN USA

**Keywords:** animal models, cerebral palsy, mice, reproduction, survival rate

## Abstract

Characterization of growth and survival of mice displaying early onset hypertonic symptoms is critical as these animals are important for research investigating mechanisms and treatments of pediatric conditions associated with hypertonia, such as cerebral palsy. Currently, most animal models of cerebral palsy reproduce risk factors for developing this condition, with most failing to develop the physical symptoms or failing to survive in the postnatal period. The B6.Cg‐*Glrb^spa^*/J (Gly receptor mutation) transgenic mouse (*spa* mouse), displays symptoms of early onset hypertonia, though little has been reported on growth and survival, with no reports of growth and survival since genotyping became available. We found that the majority of *spa* mice display symptoms by P14‐P16. Of mice surviving to weaning, only ~9% were *spa* mice. By weaning age, *spa* mice had significantly lower weights than their heterozygote and wild‐type littermates. Of mice that died after weaning and prior to use in experiments or being culled, 48% were *spa* mice. The poor growth and decreased survival of *spa* mice across multiple developmental and adult ages resembled the varied survival rates observed in humans with mild or severe cerebral palsy. The understanding of the expected survival of these mice is helpful for planning breeding and animal numbers for experiments. Due to the symptoms and timing of symptom onset, *spa* mice will be valuable in uncovering mechanisms and long‐term effects of early onset hypertonia in order to move toward interventions for these conditions.

## 1 INTRODUCTION

Over the years there has been the emergence of a variety of spontaneous glycine (Gly) receptor mutations in c57 black 6 mice. One of these, the B6.Cg‐*Glrb^spa^*/J (Gly receptor mutation) transgenic mouse (*spa* mouse), displays early onset hypertonia (spasticity).^1^, ^2^ *Spa* mice have a homozygous insertion of LINE‐1 in the beta subunit of the Gly receptor gene resulting in a splicing error of this subunit.^3^ This autosomal recessive mutation affects glycine receptors in both the brain and spinal cord.^2^, ^4^ Mice that have mutations in both sets of genes are termed “*spa*” mice, short for *spastic*. Since the recognition and original description of this mouse in 1961, most work has focused on the glycine receptor abnormality with little attention paid to the growth and developmental or onset of symptoms of *spa* mice.^1^, ^2^, ^3^, ^4^

Characterization of growth and survival of these mice is critical as animals displaying early onset hypertonia are important for research investigating mechanisms and treatments of pediatric conditions associated with hypertonia, such as cerebral palsy. Currently, most animal models of cerebral palsy are based on reproducing risk factors for developing this condition, with most animal models failing to develop the physical symptoms.^5^ For conditions of early onset hypertonia, such as cerebral palsy, surprisingly little is known about the mechanisms underlying the development of hypertonia.^5^ With the NIH encouraging pursuit of animal models for the study of cerebral palsy, a greater understanding of these mice and their survival is important.^6^ To study the mechanisms underlying early onset hypertonia, animals which consistently display the appropriate symptoms are crucial. Very few animals display a hypertonic phenotype that emerges in the developmental period with husbandry of these animals often being complex and survival poor.^5^ Therefore, longitudinal reporting of the growth and survival of *spa* mice is important for planning studies involving these animals while also respecting the 3Rs (replacement, reduction, and refinement) of animal research.

## 2 MATERIALS AND METHODS

### 2.1 Animals and breeding

The mouse colony was bred, maintained, and underwent experimental procedures under the Institutional Animal Care and Use Committee at Mayo Clinic (Protocols #A23215‐15, A00003598‐18, A00003622‐18) which is in compliance the American Veterinary Medical Association, US National Research Council's Guide for the Care and Use of Laboratory Animals, and US Public Health Service Policy on Care and Use of Laboratory Animals.^7^, ^8^, ^9^ B6.Cg‐*Glrb^spa^*/J mice (C57 background) were obtained from Jackson Laboratories (Jax stock #000066; Bar Harbor, ME, USA) in 2015. Due to mice that are homozygous for the mutation (*spa* mice) having impaired ability of sperm to fertilize an egg^10^ and concern for *spa* mouse females ability to rear pups due to physical symptoms, a heterozygote × heterozygote breeding scheme was used with matings starting when animals reached ~3 months of age and continuing until ~6 months of age (usually allowing for 3‐5 litters). At 2 time points, c57 mice were purchased from Jackson Laboratories for mating with a heterozygote mouse from the colony (2 pairs) with 1 or 2 litters per pair. The heterozygote mice offspring from these matings were then used for breeding. The mouse colony was tracked using the SoftMouse (Toronto, Ontario, CA) online digital platform.

### 2.2 Animal genotyping

Genotyping was performed from a 2‐ to 5‐mm tail snip obtained at the time of weaning of pups or after euthanasia of fetuses and pups of preweaning age. Genotypes were determined by PCR using the following primers. Wild‐type forward*, 5′‐GCAACTTGAGAGC‐TGTATGT‐3′*, and wild‐type reverse, *5′‐ACTTGGCTGGGCTTACATAT‐3′*; wild‐type allele, 348 bp; *spa* forward, *5′‐TTCCTAAGTTCCGGT‐CTGTG‐3′*, and *spa* reverse, *5′‐CAATTATCAAGGCTGATGGC‐3′*; *spa* allele, 358 bp.^11^, ^12^ Mice who had only wild‐type alleles were identified as “wild type.” Mice that had both a wild‐type allele and *spa* allele were identified as “heterozygotes.” Mice that had only *spa* alleles were identified as “*spa*.”

### 2.3 Housing and husbandry

Mice were housed in identical conditions, following recommended housing and care guidelines,^7^ in a pathogen‐free facility and in an area separate from other mice colonies. Mice were exposed to 12:12 hours light:dark cycle year round. The room was kept at ~21‐23°C. Cages were cleaned and changed weekly by veterinary technicians. Mouse chow (PicoLab^®^ Rodent Diet 5053, LabDiet, St. Louis, MO) and tap water (via water bottles) were freely accessible. For mated pairs, breeder chow (PicoLab^®^ Rodent Diet 5058, LabDiet, St. Louis, MO) was provided. Weaning of pups was performed between 21 and 28 days of age. Litters were not culled, regardless of size, due to lack of *spa* phenotype at birth. At weaning, if a pup displayed more severe spastic symptoms (ie, spasms that required manually placing animal in an upright position or disturbance by another mouse elicited symptoms), Boost Diet Gel^®^ (Portland, ME) was made available on the cage floor and the animal was housed individually. Social housing (up to 5 mice of same sex and litter) was utilized for all other mice. All mice were housed in Jag 75 (Allentown, Inc, Allentown NJ) cages with 484 cm^2^ of floor area. For cages with *spa* mice with severe phenotypes, a paper mat and preconfigured nesting material was placed in the cage. For all other mice, including breeder pairs, standard shavings with paper material used for nesting were provided.^13^

### 2.4 Animal weights

Animals used for experimental purposes were weighed prior to experimental procedures. Age groups were selected based on generalized grouping including preweaning mice (P14‐P16), weaning to 3 months of age (immature adult), 3‐6 months of age (early mature adult), and >6 months of age (late mature adult to old).^14^ All animals were weighed using Entris Top Loading Scale (Sartorius Lab Instruments, Goettingen, Germany).

### 2.5 Animal survival

Mice in the colony were not bred for survival studies, but rather for specific experiments, which were performed at ages targeting key developmental times^15^ or at mature adult stages (~3 to 8 months old).^16^, ^17^ Mice not needed for experiments or breeding were euthanized following confirmation of genotype (~6 to 8 weeks of age) in accordance with American Veterinary Medical Association guidelines.^8^ Genotypes were determined by PCR,^11^, ^12^ with mice designated as wild type, heterozygote, or *spa* depending on the expression of wild‐type and/or *spa* alleles. Animals were weighed with Entris Top Loading Scale (Sartorius Lab Instruments, Goettingen, Germany) prior to experimental procedures. Ages of animals were selected based on developmental time points of interest for planned experiments including preweaning mice (P14‐P16), weaning to 3 months of age (immature adult), 3‐6 months of age (early mature adult), and >6 months of age (late mature adult to old).^14^, ^15^

### 2.6 Data analysis and statistics

All statistical analyses were performed using Prism 7.0 (Graphpad, La Jolla, CA). With respect to continuous variables, differences between groups were examined using unpaired t‐tests when data were normally distributed according to D’Agostino and Pearson normality tests. Two‐way ANOVA was used when comparing 2 factors, with Bonferroni *post hoc* tests where appropriate. Chi‐squared tests were used for evaluating relationships between variables. Statistical significance was established at the *P* < .05 level. All experimental data are presented as mean ± 95% confidence intervals, unless otherwise specified.

## 3 RESULTS

### 3.1 Animal survival

**FIGURE 1:**
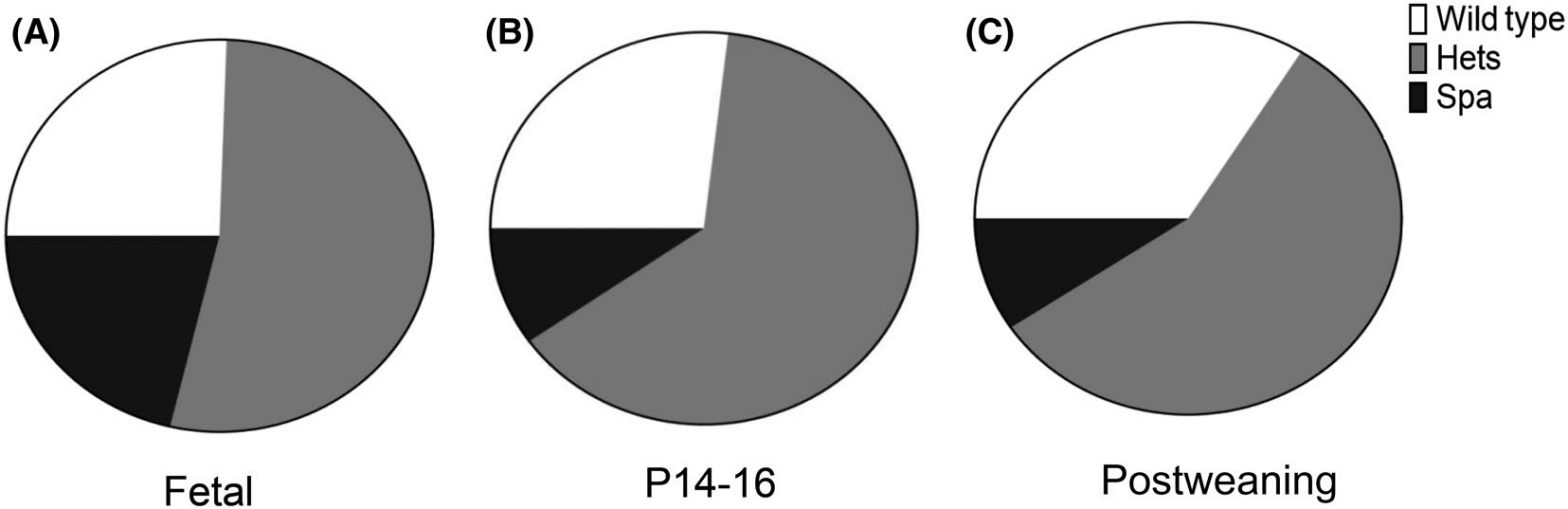
Proportion of mice based on genotypes. Proportion of mice with wild‐type (white), heterozygote (gray), and *spa* (black) genotypes in the (A) fetal, (B) ~ 2 weeks postnatal, and (C) postweaning. Based on Mendelian inheritance pattern, it would be expected that ~25% of mice would be wild type and 25% *spa* with 50% heterozygotes. This similar pattern is seen in the fetal mice with 21% being *spa* mice. However, by P14/P16, the percentage of spa mice decreased to 10% with relative expansion of heterozygote mice percentage. In the postweaning period, percentage of *spa* mice had a slight decrease to about 9% with a relative expansion of both heterozygotes (57%) and wild‐type (34%) mice percentages

To assess the influence of genotype on mouse attrition prior to weaning, litter numbers were recorded for 105 litters. The mean litter size across 105 litters was 6 (range 1‐12) pups. With regard to the fetal mice (embryonic day (E) 15‐E18), a total of 47 mice from 6 litters were euthanized for experimental purposes. Genotyping of the fetal mice (E15‐E18) revealed a roughly Mendelian distribution of genotypes as expected from a heterozygote by heterozygote breeding scheme, 10 (21.3%) mice were *spa*, 25 (53.2%) mice were heterozygotes, and 12 (25.5%) mice were wild type (Figure 1A). Of the P14‐P16 mice, 8 (9.8%) were *spa*, 52 (63.4%) were heterozygotes, 22 (26.8%) were wild type (Figure 1B). Approximately 67% of *spa* mice had the hypertonic phenotype by P14‐P16. All *spa* mice displayed a hypertonic phenotype by weaning which persisted. No *spa* mice had spontaneous resolution of hypertonic symptoms. Of mice that survived weaning, 71 (9.4%) were *spa*, 417 were heterozygotes (56.7%), and 249 (33.9%) were wild type (Figure 1C). Overall, these results show a remarkable attrition in *spa* mice compared to both wild‐type and heterozygote mice during postnatal maturation. Between birth and P14‐16 only ~40% of *spa* pups survive, with minimal additional attrition by weaning. The reduced survival of *spa* mice compared to both wild‐type and heterozygote mice was considered statistically significant (Chi‐square, *P* = .0048 at P14‐16 and *P* < .0001 at weaning).

For the 735 mice that survived weaning, 40 (5.4%) mice died of causes unrelated to experiments or being culled for the colony. Of these 40 mice, 19 were *spa* (47.5%), 17 were heterozygotes (42.5%), and 4 were wild type (10.0%). The increased likelihood of death of *spa* mice compared to heterozygote and wild‐type mice was considered statistically significant (Chi‐square, *P* < .0001).

### 3.2 Body weight gain

**FIGURE 2:**
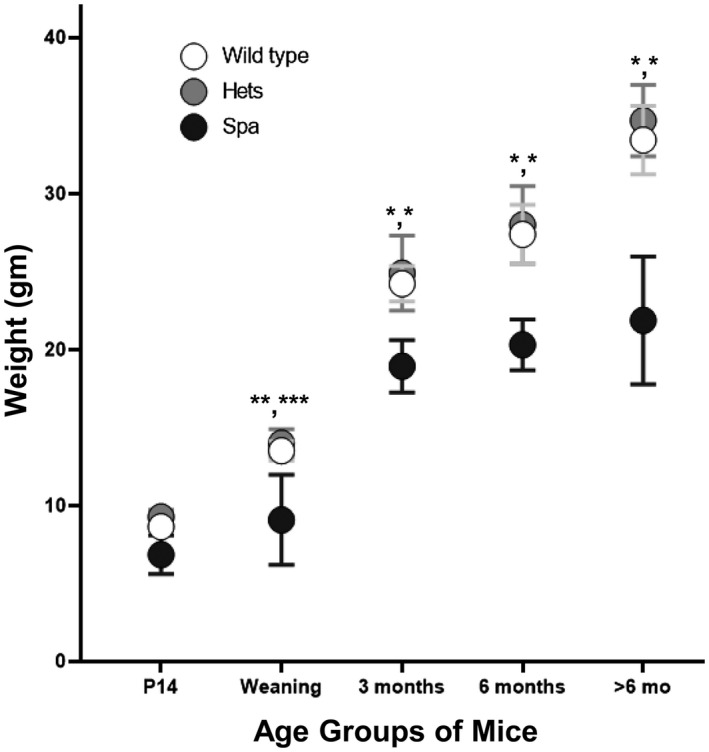
Body weights of mice based on age and genotype. *Spa* mice (black) had a significantly lower body weight at all time points postweaning as compared to wild‐type (white) and heterozygote (gray) mice (mean ± CI, Two‐way ANOVA with *post hoc* Bonferroni testing, *P < .0001, **P = .034 (spa vs wild type), ***P = .015 (spa vs heterozygote)). There was no significant difference between body weights of heterozygote and wild‐type mice at each time point

Body weights were obtained when mice were used for experimental purposes or routinely at ages from P14 through approximately 1 year of age. At all ages, *spa* mice had lower weights when compared to heterozygote and wild‐type mice, with this difference being statistically significant at all time points from weaning onward (Table 1, Figure 2). There was no significant difference in body weight between heterozygote mice and wild‐type mice at any time point (Table 1, Figure 2). When comparing body weights of *spa* mice across ages, the only significant increase in body weight compared to the previous weight occurred between weaning and 3 months of age (*P* < .0001, Table 1).

**Table 1:** Mice weight for age and genotype

Age	Genotype	Body weight (g)	*P* value vs wild type	*P* value heterozygote vs *Spa*	*P* value of same genotype vs previous age
P14‐P16	Wild type	8.6 (±0.6)	—	—	—
	Heterozygote	9.3 (±0.5)	*P* > .999	—	—
	*Spa*	6.9 (±1.2)	*P* > .999	*P* = .495	—
Weaning	Wild type	13.5 (±0.6)	—	—	*P* = .012
	Heterozygote	14.0 (±0.9)	*P* > .999	—	*P* = .0001
	*Spa*	9.1 (±2.9)	*P* = .034	*P* = .015	*P* > .999
Postweaning to <3 mo	Wild type	24.2 (±1.1)	—	—	*P* < .0001
	Heterozygote	24.9 (±2.4)	*P* > .999	—	*P* < .0001
	*Spa*	18.9 (±1.7)	*P* < .0001	*P* < .0001	*P* < .0001
3 mo to <6 mo	Wild type	27.4 (±1.9)	—	—	*P* = .007
	Heterozygote	28.0 (±2.5)	*P* > .999	—	*P* = .203
	*Spa*	20.3 (±1.6)	*P* < .0001	*P* < .0001	*P* > .999
≥6 mo	Wild type	33.4 (±2.2)	—	—	*P* < .0001
	Heterozygote	34.7 (±2.3)	*P* = .872	—	*P* < .0001
	*Spa*	21.9 (±4.1)	*P* < .0001	*P* < .0001	*P* > .999

Note

Mean (CI) weight of mice by genotype across ages. There was no significant difference in body weights of all mice at postnatal day 14‐16 (P14‐P16). However, at all ages from weaning onward, *spa* mice weighed significantly less than wild‐type and heterozygote mice. There was no significant difference in body weights between wild‐type and heterozygote mice at each age. When comparing body weights across ages within genotypes, weight gain occurred as age increased. For wild‐type mice, body weight increased significantly at all ages. For heterozygote mice, body weight increased significantly at all ages, except between the ages of postweaning to <3 mo and 3 mo to <6 mo. For *spa* mice, the only significant increase in body weight occurred between weaning and postweaning to <3 mo of age. Two‐way ANOVA with *post hoc* Bonferroni testing.

As expected, at every time point assessed, female mice had significantly lower body weights than male mice (data not shown). At each age, there were no differences in body weights between female heterozygote and wild‐type mice nor between male heterozygote and wild‐type mice (Table 2). From 3 months of age and older, female *spa* mice weighed significantly less than both heterozygote and wild‐type female mice (Table 2). In contrast to females, male *spa* mice weighed significantly less than both male heterozygote and wild‐type mice for all postweaning ages assessed (Table 2).

**Table 2:** Mice body weights across postweaning ages for males and females

Age	Sex	Wild‐type Body weight (g)	Heterozygote Body weight (g)	*Spa* Body weight (g)	*P* value wild type vs *Spa*	*P* value heterozygote vs *Spa*
Postweaning to <3 mo	Female	20.6 (±0.6)	20.8 (±2.2)	18.3 (±2.0)	*P* > .999	*P* > .999
	Male	26.9 (±0.7)	29.0 (±1.6)	19.4 (±3.0)	*P* < .0001	*P* < .0001
3 mo to <6 mo	Female	23.7 (±1.0)	25.2 (±2.39)	18.0 (±1.6)	*P* < .0001	*P* < .0001
	Male	32.7 (±2.1)	32.7 (±1.7)	22.5 (±2.4)	*P* < .0001	*P* < .0001
≥6 mo	Female	30.4 (±4.1)	32.4 (±3.44)	20.6 (±4.4)	*P* < .0001	*P* < .0001
	Male	35.4 (±2.9)	36.6 (±3.1)	26.8 (±10.5)	*P* = .0013	*P* = .0003

Note

Mean (CI) body weight of mice by sex and genotype across mature ages. At all ages postweaning in males and over 3 mo of age in females, *spa* mice weighed significantly less than wild‐type and heterozygote mice of the same sex. Wild‐type and heterozygote mice of the same sex and age had no significant difference in body weight. Two‐way ANOVA with *post hoc* Bonferroni testing.

## 4 DISCUSSION

Little has been reported on growth and survival of *spa* mice since they were originally described in 1961,^2^ which was prior to availability of genotyping.^18^ Subsequently, the specific genetic abnormality for *spa* mice was identified in 1994.^18^ Despite the phenotypic reevaluation of *spa* mice in conjunction with genotyping in 1997,^4^ the growth and survival of *spa* mice was not reevaluated. Furthermore, despite the cerebral palsy‐like phenotype of *spa* mice which prompted their use in sentinel work on botulinum toxin in hypertonic muscles,^19^ use of these mice for exploring the developmental changes in the central nervous system and motor units in conditions of early onset hypertonia remain virtually unexplored.

In *spa* mice, we identified that: (a) there is a remarkable attrition prior to weaning and before the phenotypic onset; (b) the majority of *spa* mice have a *spa* phenotype detectable at P14‐P16, prior to previously described onset at ~P21; and (iii) the developmental body weight gain of *spa* mice is impaired compared to wild‐type and heterozygote littermates. These observations have important implications for experimental design considerations in *spa* studies, including humane endpoints for body weight reduction.^20^

*Spa* mice have the highest attrition between birth and P16. This may be related to maternal difference in rearing of the mutant pups, though maternal‐pup interactions were not evaluated as part of this study.^21^ In mice with a genetic mutation resulting in a lack of gephyrin (a protein required for Gly receptor clustering) death occurs by P1 due to failure to suckle with these pups showing a rapid development of hyperextension limb posturing prior to death.^22^ It is also possible that *spa* mice pups have difficulty with suckling, as *spa* mice have an alteration in Gly signaling. In our study, all homozygous *spa* mice exhibited the spastic phenotype by P28, with a majority displaying a detectable phenotype by ~P14‐16. Although we did not evaluate ventilation in these mice, hypertonic activation of the diaphragm or upper airway muscles may result in an apnea. The frequency and duration of apneic events in *spa* mice younger than P14 and older than P14 will be a key indicator of whether respiratory dysfunction is the cause of *spa* deaths prior to weaning. It may be the case that a fraction of *spa* mutants are symptomatic before weaning and die before overt signs are readily observable. Additional loss of *spa* mice occurred postweaning with the number of incidental deaths compared to heterozygote or wild‐type mice being disproportionately greater in *spa* mice. This survival difference in the postweaning period has not been explicitly evaluated.^4^ Death of *spa* mice has notable implications for estimating colony breeding and number of *spa* mice needed for experiments, particularly if older *spa* mice are needed. While death of *spa* mice is high, this corresponds with death in inhuman conditions of early onset hypertonia, like cerebral palsy, where mortality rates before age 2 years is up to 10% and mortality by maturity up to 60%, depending on the severity of symptoms.^23^, ^24^

S*pa* mice show a significant impairment in body weight gain across all ages postweaning. This observation was consistent in both female and male mice, though the pattern of body weight gain differed between males and females. This impaired body weight gain makes *spa* mice distinctly smaller than their heterozygote and wild‐type sex‐matched littermates, different than previously reported.^4^ It is not clear if this difference is due to the *spa* mice physical symptoms alone, impaired feeding, or if there is some maternal stress in rearing of *spa* pups that contributes to their lower body weight.^25^ Interestingly, the impaired growth of *spa* mice mirrors the lower growth rates observed clinically in conditions of early onset hypertonia, like cerebral palsy. Specifically, in children with cerebral palsy, the differences in weight and height as compared to age‐matched peers are more pronounced with age, with children with cerebral palsy being lighter and shorter than their age‐matched peers.^26^

We know that by adulthood, *spa* mice have reduced motor neuron numbers in a variety of motor pools^16^, ^17^ and a reduced response of muscle to neuromuscular transmission (ie, neuromuscular transmission failure).^27^, ^28^ Current efforts within the laboratory are aimed at determining the developmental time course of motor neuron loss and muscle weakness in relation to symptom onset (P14‐28) and weaning (P28). Other groups have established that both motor neurons and the associated spinal interneuronal network exhibit reduced glycinergic inhibition in 2‐ to 6‐week‐old mice.^11^, ^29^, ^30^, ^31^ Importantly, the pathology of *spa* mice may be a contingent on the transition from the depolarizing to hyperpolarizing effect of chloride channel activation in response to glycine and/or GABA that occurs prior to P14 in rodents.^32^ The present work, establishing the gross phenotype, weight, and survival throughout development of the *spa* mice, will enable us to rationalize the experimental design and timeline to assess the motor system‐ and molecular‐level pathology. This critical work is needed for furthering the understanding of potential mechanisms underlying human conditions of early onset hypertonia. Thus, understanding growth and survival of *spa* mice is important for investigators and animal care facilities working with these or similar genetically modified mice. Provision of appropriate husbandry of these animals and the accurate estimation of survival for adequate experimental animal numbers is essential for rigorous, robust, and repeatable experiments.

## CONFLICT OF INTEREST

The authors declare that there is no conflict of interest.

## AUTHOR CONTRIBUTIONS

JEB, MJF, and GCS contributed to the design of the study, the interpretation of the data, drafting, and critical revisions of the manuscript. MJF and JEB also contributed to the acquisition of the data. All the authors approved the final version of the manuscript.
